# Excessive left ventricular trabeculation does not promote cardiac dysfunction in asymptomatic middle aged and older individuals with preserved cardiac function: an analysis from the Multi-Ethnic Study of Atherosclerosis

**DOI:** 10.1186/1532-429X-17-S1-O25

**Published:** 2015-02-03

**Authors:** Filip Zemrak, Mark A Ahlman, Gaby Captur, Saidi A Mohiddin, Nadine Kawel-Boehm, Martin R Prince, James Moon, Gregory Hundley, Joao A Lima, David Bluemke, Steffen E Petersen

**Affiliations:** 1 Centre of Advanced Cardiovascular Imaging, Queen Mary University of London/The London Chest Hospital, London, UK; 2 Radiology and Imaging Sciences, National Institutes of Health Clinical Center, Bethesda, MD, USA; 3 MRI Unit, The Heart Hospital, London, UK; 4 Institute of Cardiovascular Science, University College London and The Heart Hospital, London, UK; 5 Department of Radiology, Kantonsspital Graubuenden, Chur, Switzerland; 6 Weill Cornell Medical Center, New York, NY, USA; 7 Department of Internal Medicine, Wake Forest University, Winston-Salem, NC, USA; 8 Cardiology Division, Department of Medicine, Johns Hopkins Hospital, Baltimore, MD, USA

## Background

Left ventricular (LV) trabeculation is highly variable between individuals, and prominent trabeculation, or non-compaction, has been considered to be evidence of abnormal cardiac development. The significance of increased trabeculation in asymptomatic healthy individuals with preserved LV systolic function is unknown. The aim of this study was to determine if excessive LV trabeculation in middle aged and older subjects without LV dysfunction or advanced cardiac disease was associated with changes in cardiac volumes and function over the ensuing 10 years.

## Methods

Asymptomatic individuals (n=2711, mean age 68.7 years, 52.8% women, 56.3% with hypertension, 16.7% with diabetes) from the Multi-Ethnic Study of Atherosclerosis with preserved systolic function at baseline cardiac magnetic resonance (CMR) were assessed. The extent of trabeculation, expressed as the ratio of non-compacted to compacted (NC/C) myocardium was measured on long-axis cine images in the second CMR study 9.5 years after the baseline scan. NC/C ratios were considered in quintiles with top quintile values exceeding published normal thresholds (2.46 - 5.41). We examined associations between maximal NC/C ratio and changes of (body surface area) indexed end-systolic volume (ESVi), indexed end-diastolic volume (EDVi) and ejection fraction (EF) over 9.5 years.

## Results

Maximum NC/C ratio ranged from 0 to 5.41. Over 9.5 years, ESVi decreased by 1.3 ml/m^2^, EDVi decreased by 5.1 ml/m^2^ and EF decreased by 0.7% (p<0.0001 for all parameters).

Increase in NC/C ratio by one unit (e.g. 1 to 2) was associated with 1.1 ml/m^2^ increase in ESVi, 2.7 ml/m^2^ increase in EDVi and no change in EF when adjusted for demographic data, risk factors and baseline LV parameters. The changes are clinically irrelevant.

**Figure 1:**
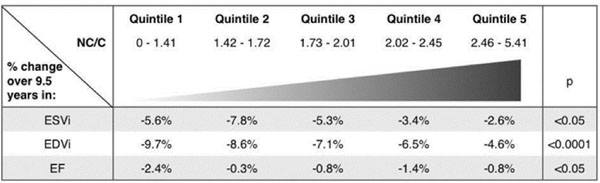
Changes in end-systolic index (ESVi), end-diastolic volume index (EDVi) and ejection fraction (EF) over 9.5 years by quintiles of maximal non-compaction ratio (NC/C).

Changes in ESVi, EDVi and EF between quintiles of NC/C ratio are presented in Figure 1. There were no clinically relevant differences in LV volumes and systolic function change between quintiles, even in subjects with excessive trabeculation.

## Conclusions

The severity of LV trabeculations in middle aged and elderly asymptomatic individuals without LV dysfunction, advanced coronary artery or significant valvular heart disease is not associated with deterioration in LV volumes or function over the ensuing 10 years. Long-term follow-up of such individuals may not be warranted.

## Funding

National Institute for Health Research Cardiovascular Biomedical Research Unit at Barts (FZ, SEP) and National Institute of Health (MESA).

